# Multi-label classification to predict antibiotic resistance from raw clinical MALDI-TOF mass spectrometry data

**DOI:** 10.1038/s41598-024-82697-w

**Published:** 2024-12-28

**Authors:** César A. Astudillo, Xaviera A. López-Cortés, Elias Ocque, José M. Manríquez-Troncoso

**Affiliations:** 1 Computer Science Department, Engineering Faculty, Universidad de Talca, Talca, Chile; 2 Department of Computer Sciences and Industries, Universidad Católica del Maule, Talca, Chile; 3 Centro de Innovación en Ingeniería Aplicada (CIIA), Universidad Católica del Maule, Talca, Chile

**Keywords:** Biomarkers, Diagnostic markers, Predictive markers, Biological techniques, Analytical biochemistry, Bioinformatics, Mass spectrometry, Proteomic analysis, Computational biology and bioinformatics, Data acquisition, Data integration, Data mining, Machine learning

## Abstract

Antimicrobial resistance (AMR) poses a significant global health challenge, necessitating advanced predictive models to support clinical decision-making. In this study, we explore multi-label classification as a novel approach to predict antibiotic resistance across four clinically relevant bacteria: *E. coli*, *S. aureus*, *K. pneumoniae*, and *P. aeruginosa*. Using multiple datasets from the DRIAMS repository, we evaluated the performance of four algorithms – Multi-Layer Perceptron, Support Vector Classifier, Random Forest, and Extreme Gradient Boosting – under both single-label and multi-label frameworks. Our results demonstrate that the multi-label approach delivers competitive performance compared to traditional single-label models, with no statistically significant differences in most cases. The multi-label framework naturally captures the complex, interconnected nature of AMR data, reflecting real-world scenarios more accurately. We further validated the models on external datasets (DRIAMS B and C), confirming their generalizability and robustness. Additionally, we investigated the impact of oversampling techniques and provided a reproducible methodology for handling MALDI-TOF data, ensuring scalability for future studies. These findings underscore the potential of multi-label classification to enhance predictive accuracy in AMR research, offering valuable insights for developing diagnostic tools and guiding clinical interventions.

## Introduction

Antibiotic resistance is considered a global health problem, with the World Health Organization (WHO) warning that unless urgent action is taken, a “post-antibiotic era” could be faced, where common infections and minor injuries can become deadly^1^. In the United States alone, over 23,000 deaths per year are attributed to this cause^2^. Among the predominant factors contributing to the increase of antimicrobial resistance (AMR), three clear culprits can be identified: the indiscriminate prescription by doctors worldwide^3^, indiscriminate use in livestock^4^, and environmental factors that facilitate the distribution of resistant genes^5^. Antibiotic resistance occurs when organisms such as bacteria, viruses, fungi, and parasites evolve and adapt to continue their development in the presence of drugs that previously affected them^6^. AMR can be classified into two categories: intrinsic resistance, where specific bacterial gender or species have unique structural or functional characteristics that confer resistance to certain antibiotics, such as *Mycoplasma spp*., which, due to the lack of a cell wall, is resistant to beta-lactam and glycopeptide antibiotics^7^. Moreover, acquired resistance involves naturally susceptible bacteria that develop antibiotic resistance through the adaptation of the genetic code of resistant strains, which can occur through mechanisms, including i) modification or inactivation of antimicrobial agents by enzymes^8^, ii) reduction of intracellular accumulation of antimicrobial agents^7^, and iii) alterations in the target sites of antimicrobial agents^9^. The clinical routine for selecting the best therapeutic option for the treatment of bacterial infections relies on the results obtained from antimicrobial susceptibility testing (AST)^10^. Currently, there are several methods of AST, but the most conventional and widely used involves extracting a bacterial sample for culture and observing its growth in the presence of a specific antimicrobial, a process that can take up to 72 hours after species identification. Application of machine learning (ML) for Mass Spectrometry (MS) data has been explored in different fields in order to predict and identify bacteria and diseases^11–15^. Matrix-Assisted Laser Desorption/Ionization Time-Of-Flight (MALDI-TOF) Mass Spectrometry technique has been adopted to characterize the protein composition of bacteria and fungi^16^, enabling rapid, accurate, and cost-effective species identification. MALDI-TOF is an analytical technique used to identify and characterize molecules, particularly proteins, by measuring their mass-to-charge ratio. In this method, a sample is mixed with a matrix material and subjected to a laser pulse, causing the ionization and desorption of the sample into the gas phase. The ions are then accelerated in an electric field and their time of flight is measured, which is used to determine the mass of the molecules. MALDI-TOF is widely used in microbiology for identifying microorganisms and in proteomics for analyzing protein samples^17^.

The use of machine learning (ML) coupled with post-analysis of MS data using supervised learning algorithms such as Support Vector Machines (SVM), Naive Bayes (NB) and Random Forest (RF) among others, has played a significant role in subspecies identification^18–22^. In other cases, deep learning (DL) has been employed to identify over 1000 bacterial species from MALDI-TOF mass spectra^23^.

For the study of antimicrobial resistance, research has focused on bacteria and antibiotics prioritized by the World Health Organization (WHO), and it has been shown that MALDI-TOF provides detailed proteomic information that allows the identification of antibiotic resistance biomarkers^24,25^. In specific, for the case of antibiotic resistance, a variety of studies have been applied, for example, the identification of Vancomycin-susceptible *Enterococcus faecium* by employing RF, SVM, and KNN (K-Nearest Neighbors) algorithms^26^. Other studies have combined mass spectra of *Staphylococcus aureus* strains with demographic characteristics such as age and sex to improve Logistic Regression (LR) algorithms^27^. Regarding the optimization of mass spectra to improve the results of classification algorithms, various methods have been tested, ranging from the implementation of kernels to obtain *m/z* peaks with greater differentiation between classes^28^, to the use of neural networks to reduce the dimensionality of spectra through autoencoders^29^ or the use of Convolutional Neural Networks (CNN) for Vancomycin resistance classification in *Enterococcus faecium* using raw mass spectra^30^. Recently, CNN and transfer learning has also been used to predict antibiotic resistance among *S. aureus*, *E. coli* and *K. pneumoniae*^15^.

Among the different bacteria of major global study and importance, Methicillin-resistant *Staphylococcus aureus* (MRSA) stands out as one of the most challenging bacteria in terms of treatment due to the multitude of multidrug-resistant phenotypes it has developed since the first MRSA case was documented in 1960^31^. MRSA has been classified as one of the second-priority pathogens by the World Health Organization^32^. Additionally, *S. aureus* exhibits resistance to different beta-lactam antibiotics such as oxacillin^33^, ceftriaxone, or cefoxitin^34^, as well as resistance to clindamycin^35^. In this context, the development of new antimicrobials and new methods for the identification of antibiotic resistance phenotypes need to be investigated. In this regard, the use of MALDI-TOF coupled with ML analysis would optimize the common 72-hour time required to obtain a resistance profile with the AST. A good ML predictor would allow the detection of antibiotic resistance from the MALDI-TOF profile, providing an immediate clinical result upon identification, reducing waiting times for results, and improving patient survival and quality of life through timely treatment.

While much of the current research focuses on identifying resistance to individual antibiotics, the challenge of multidrug resistance remains a critical and underexplored area^36,37^. Research dedicated to multi-label classification mainly focus on the classification of Multilocus Sequence (MLST), which requires additional equipment for genomic sequencing and are often too specific to the region where the bacterial samples are collected^38–40^. This highlights the need for more generalized models that can rapidly and accurately identify resistance to commonly used antibiotics, facilitating personalized treatment strategies for patients. In this way, Zhang et al.^41^ have demonstrated the potential use of multi-label classification in the context of predicting antimicrobial resistance, particularly in the case of oxacillin- and clindamycin-resistant *s. aureus* from MS samples. However, further studies and analyses are required to test the potential application of a multi-label approach to determine AMR when a bacterium is susceptible or resistant to more than one antibiotic and explore its practical applicability in clinical and research settings.

In this study, we assess the performance of multi-label classification techniques by applying a machine learning benchmark to four clinically significant bacteria *Staphylococcus Aureus*, *Escherichia coli*, *Klebsiella pneumoniae* and *Pseudomonas aeruginosa*, along with their respective AST profile. This comprehensive approach will allow us to assess the robustness and generalizability of these methods across diverse microbial contexts. Specifically, we aim to determine the efficacy of multi-label classification in predicting AMR in these bacterial species. By conducting a comparative analysis across multiple datasets, we seek to provide valuable insights into the advantages of multi-label approach over traditional single-label methods and to explore its scalability and practical applicability in real-worls clinicial and research eviroments.

The contribution of the article are the following:

- Comprehensive investigation of antimicrobial resistance prediction: Our study leverages multi-label classification to predict antibiotic resistance across four clinically significant bacteria (*E. coli*, *S. aureus*, *K. pneumoniae*, and *P. aeruginosa*), addressing the complexities inherent in multi-drug resistance scenarios.
- Comparative analysis of classification strategies: We evaluated the performance of four classification algorithms-MLP, SVC, RF, and XGB-across multi-label and single-label settings. The results provide insights into when multi-label methods offer advantages and when single-label strategies may perform better.
- External validation across independent datasets: To enhance the robustness of our findings, we conducted external validation using the DRIAMS B and C databases. This step ensures that the models generalize well across different bacterial datasets, demonstrating their practical relevance.
- Development of a reproducible methodology for MALDI-TOF data analysis: We propose a systematic approach for handling MALDI-TOF data with multi-label outputs, ensuring reproducibility and scalability. This methodology includes data preprocessing, model optimization, and performance evaluation with weighted F1 metrics.
- Addressing class imbalance: Our study examines the impact of oversampling techniques such as SMOTE-Tomek, highlighting their limitations in the multi-label context and offering recommendations on when to avoid oversampling for better performance.
- Evaluation of interpretability with SHAP analysis: We incorporate feature importance analysis using SHAP values, identifying potential biomarkers relevant to antibiotic resistance and discussing the challenges of applying SHAP to MALDI-TOF data.
- Insights for future research and clinical applications: Our work provides actionable insights into the strengths and limitations of multi-label classification for antibiotic resistance prediction, laying a foundation for future developments in rapid diagnostic tools.

## Methods

### Data

The data were collected from the public database DRIAMS^42^. This database consists of four sub-collections, DRIAMS A, B, C and D, of which DRIAMS A contains the largest number of samples, making it the primary dataset used for building the main models. It includes MALDI-TOF mass spectra of four different bacteria: *S. aureus*, *E. coli*, *K. pneumoniae*, and *P. aeruginosa*. Consequently, a separate dataset was constructed for each bacterium. Table 1 details the number of samples per bacterium as well as the types of antibiotics considered in each case. According to Table 1, the antibiotics included for *S. aureus* were oxacillin, clindamycin, and fusidic acid. For *E. coli*, the antibiotics were ciprofloxacin, ceftriaxone, piperacillin-tazobactam, and cefepime. In the case of *K. pneumoniae*, ciprofloxacin, ceftriaxone, imipenem, and meropenem are used. Finally, for *P. aeruginosa*, the antibiotics included were ciprofloxacin, imipenem, and meropenem.

**Table 1:** Number of samples per each bacterium and their respective antibiotics under study.

Bacteria	Antibiotics	Samples
*S. Aureus*	Oxacillin, Clindamycin, Fusidic acid	3531
*E. Coli*	Ciprofloxacin, Ceftriaxone, Piperacillin-Tazobactam, Cefepime	4614
*K. Pneumoniae*	Ciprofloxacin, Ceftriaxone, Imipenem, Meropenem	2776
*P. Aeruginosa*	Ciprofloxacin, Imipenem, Meropenem	2236

The bacteria under study were carefully selected because they are identified as priority pathogens by the WHO^32^. Besides, the antibiotics chosen have registered an increased prevalence of resistant strains^33,43,44^. In this way, the samples were carefully selected based on their clinical relevance and the availability of sufficient data in the DRIAMS A database, necessary for the implementation of ML models. In detail, the database DRIAMS A corresponds to MALDI-TOF MS samples that were collected from 4 different hospitals^42^.

As per the multi-label paradigm, the dataset includes multiple label columns, each containing information on individual antibiotic susceptibility. When considering a single-label, the classification problem is of binary nature, i.e., they are susceptible (S) or resistant (R). However, in conjunction, all columns labels form a classification problem in which the model is expected to predict *all* the target variables simultaneously. As detailed in Table 1 the bacteria *S. Aureus* and *P. aeruginosa* have three class labels. Besides, *E. coli* and *K. pneumoniae* possess four class labels.

### AMR workflow

**Fig. 1:**
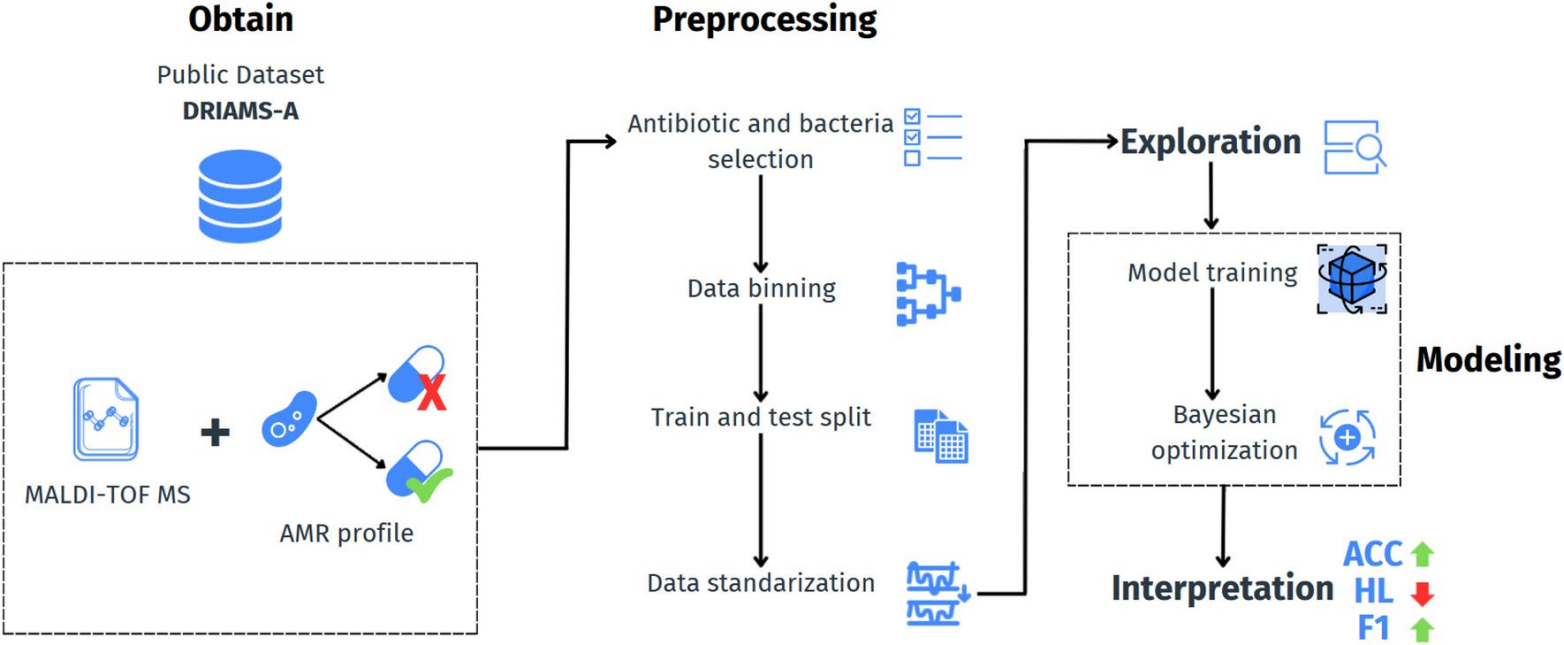
SHAP values *E. coli*-Ciprofloxacin.

The proposed workflow for studying multidrug resistance is illustrated in Fig. 1. The method adopted to classify antibiotic resistance among the bacteria consists of five key steps: obtaining, preprocessing, exploring, modeling, and interpreting the data, as detailed below. From DRIAMS A, we extracted raw mass spectra for four different bacteria *S. Aureus*, *E. coli*, *K. pneumoniae* and *P. aeruginosa*, along with their respective resistance profiles to commonly used antibiotics. Subsequently, spectra were binned into equal-sized feature vectors for analysis and modeling. Then, the dataset was split into 80% for training and 20% for testing. The correlation between the resistance profiles was analyzed, along with the distribution among the classes, to identify potential class imbalances. Two classification strategies were employed for comparison: a multi-label approach using the Label Power Set (LPS) method and a single-label approach, where one classifier was trained per bacterial species. Besides, four different ML algorithms were implemented: Support Vector Classifier (SVC), Multi-layer Perceptron (MLP), RF, and eXtreme Gradient Boosting (XGB). Finally, the performance was evaluated and compared for each algorithm, considering metrics such as Hamming Loss (HL), weighted F1 Score (WF1), and Accuracy (ACC).

#### Data preprocessing

For each bacterium under study, a dataset with their respective antibiotic resistance profile was constructed. Following the recommendation of Ref.^42^, duplicated samples were removed to reduce any bias. Accordingly, null values were removed. As suggested in^41,45^, a matrix was constructed ranging from 2000 to 9999 Da. The data were aggregated in blocks of 5 bins, resulting in 1600 columns. As a result, four datasets were constructed.

The data were standardized according to the z-score, which is detailed as follows:

1 $$\begin{aligned} z=\frac{x-\mu }{\sigma } \end{aligned}$$

where $\mu$ is the sample mean and $\sigma$ is the standard deviation per variable.

For validation purposes, the data were partitioned into train (80%) and test (20%) subsets using stratified random sampling.

The single-label columns were aggregated, building a multi-class variable. Then, a frequency table was created, and those classes represented with ten or fewer samples were considered underrepresented and therefore removed from the study. For example, when a bacterium is resistant to oxacillin but susceptible to clindamycin, this would originally be represented in two columns, the first with the value “R” and the other with the value “S”, respectively. The process combines both columns, analyzing them as “RS”. An analogous process is repeated for each data set and can be generalized to as many antibiotics as desired.

### Data exploration

This section focuses on identifying patterns within the data. During this stage, various visualization techniques were employed to facilitate the process. To aid in this, resistant instances were represented with an “R” and susceptible instances with an “S”. This section provides explanations of the constructed visualizations, with examples using the bacterium *S. aureus*. Visualizations for the other bacteria and antibiotics can be found in the supplementary material (Figures S1, S2 and S3).

#### Distribution of antimicrobial resistance profiles

The distribution of each antimicrobial resistance profile concerning the selected antibiotics was examined. This analysis provides information such as the number of instances susceptible to all antibiotics, the number resistant to all antibiotics, and those resistant to some but not others.

**Table 2:** Class distribution of AST profile in *S. aureus*.

Oxacillin	Clindamycin	Fusidic acid	Count
S	S	S	1931
R	S	S	335
S	R	S	228
R	R	S	150
S	S	R	87
R	S	R	63
R	R	R	16
S	R	R	14

Table 2 shows the distribution for *S. aureus*. This analysis reveals a significant imbalance, with the most prevalent profile (SSS) occurring approximately 138 times more frequently than the least common profile (SRR). Other bacteria exhibit similar behavior, where resistant instances consistently represent a minority (see Tables ST1, ST2, and ST3 in the Supplementary Material). Consequently, it can be inferred that accurately predicting resistant instances will pose a greater challenge.

#### Mass spectrometry graphs

Mass spectrometry graphs clearly delineate the susceptibility profiles for each antibiotic, facilitating the identification of specific mass ranges where distinguishing between resistant and susceptible instances is more achievable. This analysis offers valuable insights into the complexity of the issue.

**Fig. 2:**
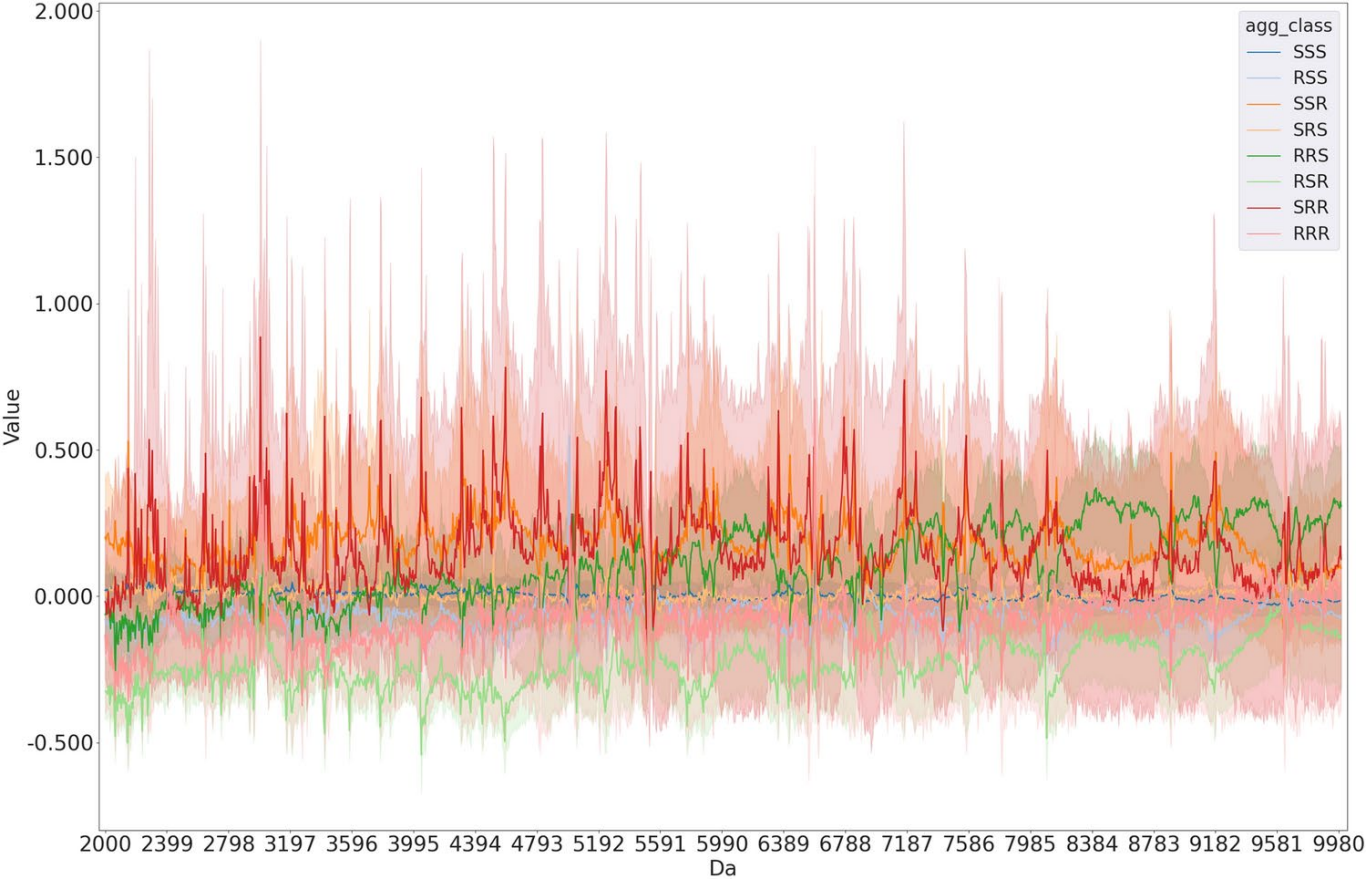
Average mass spectra for each antibiotic susceptibility combination in *S. aureus*.

Figure 2 shows the average mass spectra already standardized, along with its confidence interval, for *S. aureus*. It is observed that, on average, the behavior of the multiple classes are quite similar to one another, with the confidence intervals of the classes overlapping across almost the entire X-axis. This implies that separating one class from the other is a complex problem, requiring sophisticated models to solve. It is important to mention that the other three bacteria and antibiotic profiles showed similar patterns (Figures S1, S2 and S3 of supplementary material).

#### Scatter plots using dimensionality reduction techniques

Scatter plots created through dimensionality reduction techniques provide a novel perspective on the data, highlighting distinguishable regions within a two-dimensional space. This method offers an alternative means of evaluating the complexity of the problem. The dimensionality reduction technique used in this analysis corresponded to Principal Component Analysis (PCA)^46^.

**Fig. 3:**
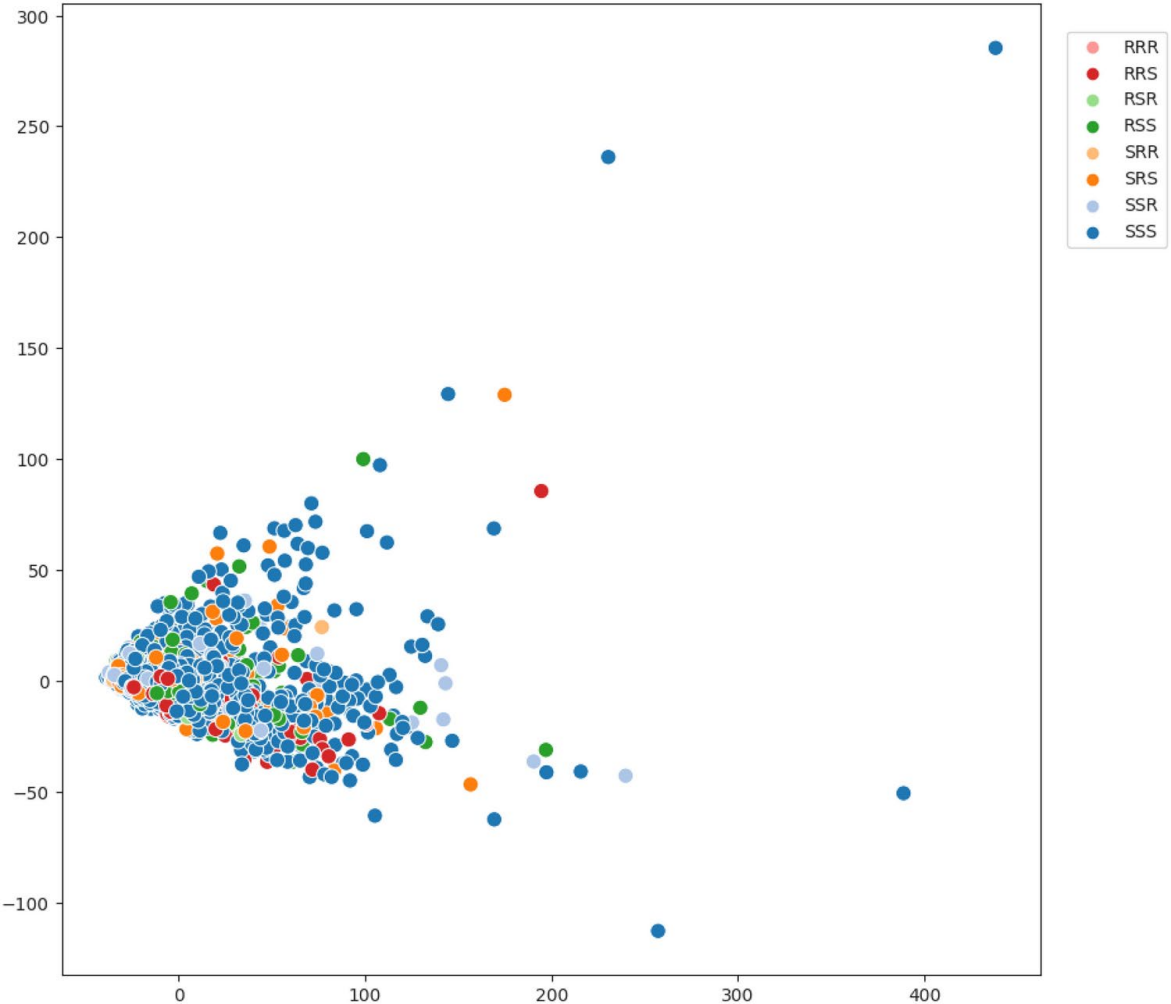
PCA for *S. aureus* and all its AMR profiles.

Figure 3 illustrates the distribution of instances in the *S. aureus* dataset visualized in a two-dimensional space using PCA. Each color in the graph corresponds to a different class, with a total of eight classes represented. Similar to the previous visualization, there is no clear separation between instances of different classes, emphasizing the challenges inherent in the classification problem. This pattern is consistent across all bacteria analyzed in this study (Figures S4, S5 and S6 of supplementary material).

### Predictive models

Four classification algorithms, eXtreme Gradient Boosting, Multi-layer Perceptron, Random Forest and Support Vector Classifier, were implemented to benchmark multi-label classification. These algorithms were selected because they belong to different families of ML methods and have demonstrated successful results in previous studies for AMR prediction^41,42,47,48^. All models were implemented in Python 3.11.5, using the package *xgboost* 2.0.3 for XGB and the package *scikit-learn* 1.2.2 for the rest.

In respect to the algorithms implemented, XGB constructs an ensemble model by aggregating predictions from multiple weak learners. This technique leverages gradient boosting to attain remarkable predictive accuracy. XGB is renowned for its ability to handle a wide range of data science tasks and is highly regarded in the field^41,49^. On the other hand, MLP is a class of feedforward artificial neural networks (ANN) that consists of multiple layers of neurons. Each neuron in a layer is connected to every neuron in the subsequent layer, creating a dense network. MLP is capable of learning complex non-linear relationships in the data through backpropagation, making it a versatile model for various tasks in machine learning^42^. Regarding RF, it constructs an ensemble model by aggregating the predictions of multiple decision trees. Each tree in the forest is trained on a random subset of the data, and the final prediction is made by majority voting. This technique enhances predictive performance and reduces the risk of overfitting, making Random Forest a robust and widely-used method in the field^42,50^. Finally, SVC seeks to identify a hyperplane that optimally separates distinct classes within the training data. SVC can also handle non-linear problems by employing kernel functions. With its versatility and robustness, SVC has found numerous applications in data science^12,42,51–53^.

In this way, XGB, MLP, RF and SVC provide robust capabilities for addressing classification challenges, making them essential tools in a data scientist’s repertoire. Furthermore, Bayesian hyperparameter tuning was performed. This strategy involves iteratively exploring the hyperparameter space to identify the optimal configuration for the models. By employing a Bayesian approach, the search process was guided by previous iterations, leading to more efficient and effective hyperparameter selection.

### Multi-label classification

Multi-label classification is a supervised learning task where each instance is associated with multiple labels simultaneously^54^. In contrast to traditional single-label classification, where each instance is assigned to only one label, multi-label classification allows instances to belong to multiple classes simultaneously. In this context, each label represents the resistance of a bacterium to a certain antibiotic. This approach allows specifying the resistance of multiple antibiotics to a single instance.

For our problem, let $\mathcal {X} = \mathbb {R}^d$ be the input space that considers *d* dimensions, and $\mathcal {Y} = \{y_1, y_2, ..., y_q\}$ denotes the label space with *q* possible class labels. Given a training dataset $\mathcal {D} = \{(\textbf{x}_1, \textbf{Y}_1), (\textbf{x}_2, \textbf{Y}_2), \ldots , (\textbf{x}_n, \textbf{Y}_n)\}$, where $\textbf{x}_i \in \mathcal {X}$ is the input feature vector associated with the *i*th instance and $\textbf{Y}_i \subseteq \mathcal {Y}$ is the set of labels associated with $x_i$, the goal of our multi-label classification task is to learn a function $h: \mathcal {X} \rightarrow 2^\mathcal {Y}$ that maps each instance to a subset of labels.

Based on this definition, binary classification of a single antibiotic is a specific case. Generally, multi-label classification is applied when there are multiple antibiotics involved.

The complexity of the problem arises from the exponential growth in combinations with the number of antibiotics. For instance, considering 20 antibiotics would result in a solution space of 2 to the power of 20, equivalent to over 1 million possible combinations^54^.

In this study, LPS was employed as an strategy to address the multi-label classification problem. LPS transforms the original multi-label task into a multi-class problem by considering each unique combination of labels as a distinct class^55^. This allows the model to capture dependencies between labels that might be missed by treating each label independently. Specifically, for each instance, the set of labels is treated as a single “power set” combination, and the classifier is trained to predict the presence of one of these combinations. While this approach effectively models correlations between labels, it increases the complexity of the problem as the number of possible combinations grows exponentially with the number of labels. Despite this challenge, LPS was selected due to its ability to better represent the interrelationships between antibiotic resistance profiles, which are often correlated across multiple antibiotics.

The Label Power Set method was selected over alternative techniques such as Classifier Chain^56^ and Binary Relevance^57^ due to its ability to capture label dependencies in multi-label classification. Unlike Binary Relevance, which treats each label independently and ignores potential correlations between them, LPS models all label combinations as distinct classes, allowing for a more accurate representation of the interrelationships between antibiotic resistances. Although, Classifier Chain partially addresses this issue by sequentially predicting labels with information from previous labels, it requires training a separate model for each label, increasing computational cost and complexity. In contrast, LPS provides a single, unified model that handles the entire label set simultaneously. Given the nature of our dataset, where resistance to multiple antibiotics is often correlated, LPS was deemed more appropriate for capturing these dependencies, ensuring a more holistic approach to predicting resistance profiles.

#### Performance metrics

To evaluate the models’ performances, several metrics were considered: ACC, HL and WF1^58^. In detail, ACC and HL were chosen due to their prevalence in previous studies^41^. However, these metrics do not account for class imbalance and may overlook the model’s effectiveness in predicting minority classes. Consequently, and considering the class imbalance observed in the databases, WF1 was selected as it emphasizes this issue more than the other metrics.

F1 was calculated for each class following Eq. (2). Then, to address class imbalance, a weighted mean was calculated, as shown in Eq. (3), resulting in the WF1. Equation (4) demonstrates how the weight of each class is calculated.

2 $$\begin{aligned} F1\,Score\,(F1)= & \frac{True\,Positives}{True\,Positives + \frac{1}{2} ({{False\,Positives}} + {{False\,Negatives}})} \end{aligned}$$

3 $$\begin{aligned} {{Weighted\,F1\,Score\,(WF1)}}= & \frac{\sum _i F1_i \times {{Weight}}_i}{\sum _i {{Weight}}_i} \end{aligned}$$

4 $$\begin{aligned} {{Weight}}_i= & \frac{{{Number\,of\,samples\,of\,class}}_i}{{{Total\,amount\,of\,samples}}} \end{aligned}$$

**Fig. 4:**
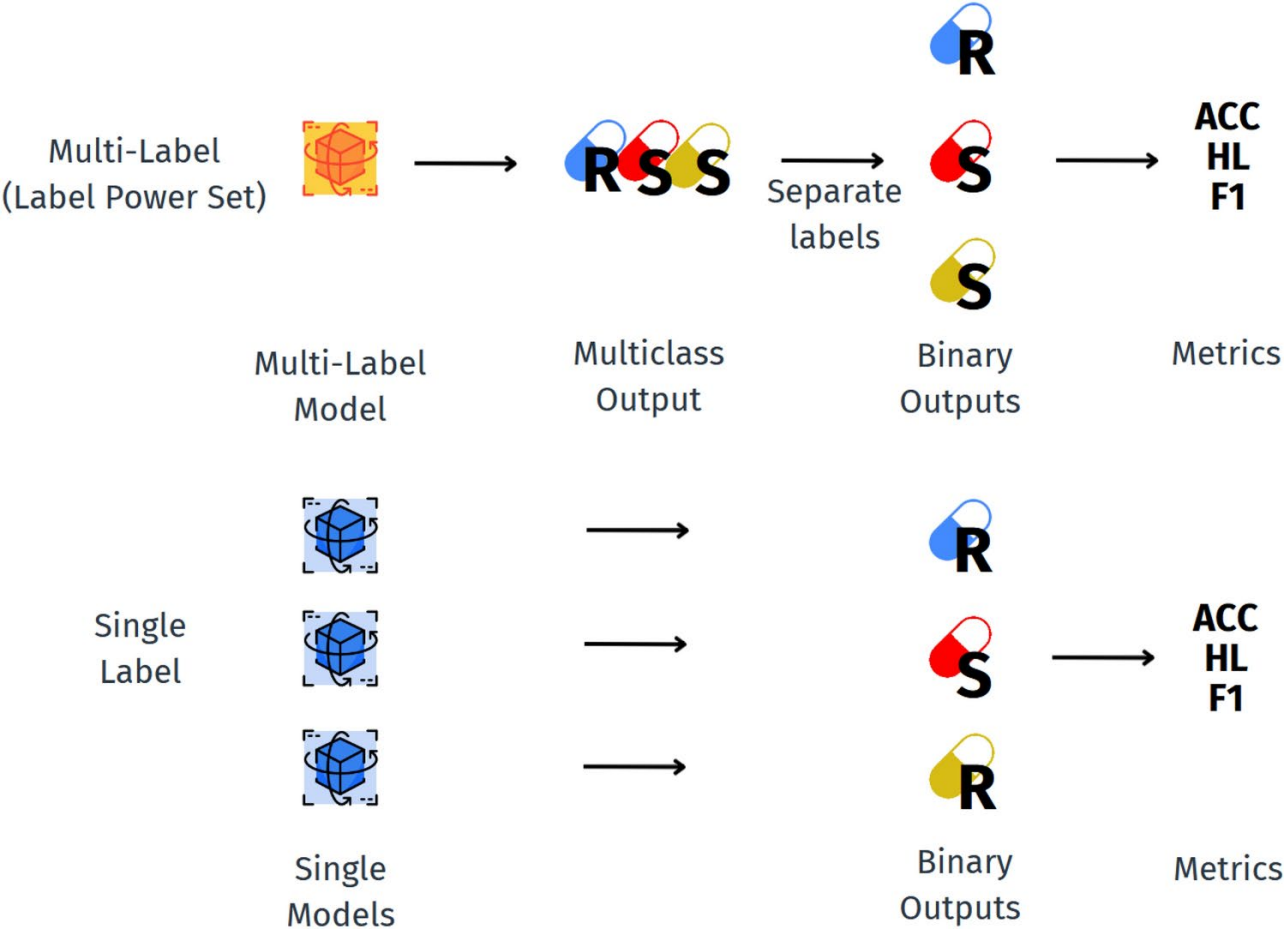
Scheme of the process used to calculate the metrics in the multi-label and single-label scenarios.

Predictions made by the models using the Label Power Set method are first transformed to multi-label before being evaluated. This situation is illustrated in Fig. 4.

For example, if the model predicts that a particular instance belongs to the class “RSS”, the prediction is divided into three independent labels. This process is illustrated in Fig. 4. As the example shows, the outputs of the single-label and multi-label models may differ. In the multi-label approach, a single model is trained to generate a single output describing the susceptibility to multiple antibiotics. Afterward, the output is separated into multiple labels. The model’s parameters are optimized, and each of these outputs is subsequently used to calculate the respective metrics (ACC, HL, WF1). Alternatively, in the single-label scheme, the same ML algorithm as in the multi-label case is employed. However, each model is optimized independently. A separate model was trained for each antibiotic, and the metrics are then calculated.

Weighted F1 score was selected as the primary metric due to its effectiveness in addressing class imbalances, which are prevalent in antibiotic resistance datasets. Unlike the standard F1 score, which treats all classes equally, WF1 assigns weights to each class based on its frequency, ensuring that underrepresented yet clinically significant classes are adequately considered when assessing model performance. This approach is especially crucial in multi-label classification tasks, such as ours, where accurate predictions across all resistance profiles are vital. By incorporating both precision and recall, WF1 offers a balanced evaluation, making it an ideal choice for assessing model performance in this context.

A rigorous evaluation approach was employed by calculating the WF1 score independently for each label. This comprehensive evaluation enables an accurate assessment of our models’ performance. Once the individual label scores are obtained, their averages are computed to derive the final results, ensuring a robust and representative evaluation. By employing this meticulous methodology, we aimed to provide a clear and reliable assessment of our model’s capabilities.

#### Parameter optimization

To enhance the quality of the results, bayesian optimization was applied to the models using the library *scikit-optimize* 0.9.0. The WF1, commonly employed in scenarios with imbalanced classes, was selected as the optimization metric. The ranges considered for each hyperparameter are detailed in Table 4 for MLP, Table 3 for RF, Table 5 for SVC, and Table 6 for XGB, providing insights into the fine-tuned configurations. All other hyperparameters not specified were set to their default values in the respective packages.

Each model was optimized separately, regardless of whether they share their underlying algorithm, bacteria, or antibiotic.

**Table 3:** Hyperparameters optimized for RF models.

Hyperparameter	Values
n_estimators	$[10^{0}, 10^{3}]$
max_depth	$[10^{0}, 10^{1}]$
min_samples_leaf	$[10^{0}, 10^{1}]$
bootstrap	$\{\texttt{False}, \texttt{True}\}$
random_state	$\{0\}$

**Table 4:** Hyperparameters optimized for MLP models.

Hyperparameter	Values
Activation	$\{\texttt{identity}, \texttt{logistic}, \texttt{tanh}, \texttt{relu}\}$
Solver	$\{\texttt{sgd}, \texttt{adam}\}$
learning_rate	$[10^{-6}, 10^{-2}]$
layer1	$[10^{1}, 5*10^{2}]$
layer2	$[10^{1}, 5*10^{2}]$
layer3	$[10^{1}, 5*10^{2}]$

**Table 5:** Hyperparameters optimized for SVC models.

Hyperparameter	Range of optimization
C	$[10^{-6}, 10^{3}]$
Kernel	$\{\texttt{rbf}\}$
Gamma	$[10^{-6}, 10^{3}]$

**Table 6:** Hyperparameters optimized for XGB models.

Hyperparameter	Values
max_depth	$[10^{0}, 10^{1}]$
min_child_weight	$[10^{-6}, 10^{1}]$
max_delta_step	$[10^{-6}, 10^{1}]$
subsample	$[10^{-6}, 10^{0}]$
tree_method	$\{\texttt{exact}, \texttt{aprox}, \texttt{hist}\}$
scale_pos_weight	$[10^{-6}, 10^{1}]$
Gamma	$[10^{-6}, 10^{1}]$
Eta	$[10^{-6}, 10^{0}]$

#### Implementation of oversampling techniques

To evaluate the impact of oversampling techniques on model performance, two methods, Random Oversampling and SMOTE-Tomek, were applied to all minority classes to achieve class balance across the dataset. The oversampling was adjusted to ensure that the number of instances in each minority class matched that of the majority class. The hyperparameters used in the models adhered to the optimization process outlined in the previous section. Performance metrics were evaluated using the WF1 score, facilitating a clear comparison between the different sampling techniques.

### External validation of the models

To evaluate the models previously trained on DRIAMS A, external validation was performed. Specifically, the DRIAMS B and C databases were used^42^, focusing specifically on *E. coli* and *S. aureus*. These two bacteria were selected because, unlike (*K. pneumoniae* and *P. aeuroginosa*), the databases incorporate the same antibiotic profiles used in the models’ implementation. For the external validation, only the models that show the best performances in the previous experiments are selected.

### Feature importance analysis with shap values

To identify the most important features associated with antibiotic resistance for each bacterium, SHapley Additive exPlanations (SHAP) values was used^59^. SHAP is a widely accepted method for interpreting complex machine learning models. SHAP values quantify the contribution of each feature to the prediction, offering a deeper understanding of the model’s decision-making process. For this study, SHAP was implemented using the shap 0.44.1 Python package, specifically with the MLP multi-label classification model. Due to the high memory demands of the SHAP explainer, we reduced the number of features by grouping them into bins of 20 Da. While configuration may result in a slight loss of information, it effectively reduces noise and has been successfully employed in prior studies^41,42^.

## Results

This section summarizes the main results obtained in this study. Table 7 presents a performance comparison among the different models MLP, RF, SVC and XGB applied to predict antibiotic resistance in *E. Coli*, *K. Pneumoniae*, *P. Aeruginosa*, and *S. Aureus*. The performance was evaluated according to the metrics of ACC, HL, and WF1, with respect to the results reported for both single-label and multi-label classification scenarios.

**Table 7:** Performance results for each model in respect to each bacteria under study.

Bacteria	Model	ACC		HL		WF1	
		Single-label	Multi-label	Single-label	Multi-label	Single-label	Multi-label
*E. Coli*	MLP	0.65	0.68	0.13	0.13	**0.87**	**0.87**
	RF	0.58	0.63	0.18	0.18	0.77	0.77
	SVC	0.66	**0.71**	0.13	**0.12**	0.86	**0.87**
	XGB	0.66	0.69	0.14	0.14	0.84	0.83
*K. Pneumoniae*	MLP	0.78	0.80	**0.07**	0.08	**0.92**	**0.92**
	RF	0.79	0.79	0.08	0.08	0.90	0.89
	SVC	0.79	0.80	**0.07**	**0.07**	**0.92**	**0.92**
	XGB	0.76	**0.82**	0.08	**0.07**	0.90	0.91
*P. Aeruginosa*	MLP	0.75	0.78	0.13	0.13	0.86	0.86
	RF	0.80	0.75	0.12	0.15	0.83	0.82
	SVC	0.77	**0.82**	0.12	**0.11**	0.86	**0.87**
	XGB	0.80	0.81	0.12	0.12	0.84	0.83
*S. Aureus*	MLP	**0.76**	0.74	**0.09**	0.11	**0.90**	0.88
	RF	0.70	0.68	0.13	0.14	0.82	0.81
	SVC	0.73	0.75	0.11	0.10	0.88	0.89
	XGB	0.72	0.72	0.11	0.12	0.86	0.86

Significant values are in bold.

### Single-label vs. multi-label comparison

The results of the single and multi-label approaches were analyzed using WF1, ACC, and HL (Table 7). Specifically, for WF1, the primary metric, the results differed between the single-label and multi-label models. Traditional single-label models outperformed in the case of *S. Aureus*, with a WF1 of 0.90 (MLP model), while the multi-label approach achieved better performance for *P. Aeruginosa*, with a WF1 of 0.87 (SVC model). For *E. Coli* and *K. Pneumoniae*, the best WF1 results were identical for both the single and multi-label approaches. For illustration, the best WF1 score for *E. Coli* was 0.87 for single and multi-label (MLP), and for *K. Pneumoniae* the WF1 was 0.92 for both approaches (MLP and SVC) (Table 7).

For ACC and HL, the multi-label models consistently yielded the best results, except in the case of *S. Aureus*. Specifically, for *E. Coli*, the best ACC and HL were 0.71 and 0.12, respectively, with an SVC model. In the case of *K. Pneumoniae*, the best results for ACC and HL were 0.82 and 0.07, achieved using an XGB model. For *P. Aeruginosa*, the best results were obtained with an MLP model, with an ACC of 0.82 and an HL of 0.11. Finally, for *S. Aureus*, the single-label approach produced better results, with an ACC of 0.76 and an HL of 0.09. However, the differences in metrics between the single-label and multi-label approaches were not statistically significant (p-value > 0.05).

#### Algorithm comparison

Throughout the experiments conducted in this study, distinct differences in performance between the single-label and multi-label approaches were observed. Additionally, the impact of oversampling methods on this type of data was thoroughly evaluated.

In respect to the algorithm comparison showed in Table 7 for the multi-label approach, the MLP model exhibited the highest WF1 across most scenarios with values of 0.87, 0.92, 0.86, 0.88 for *E. Coli*, *K. Pneumoniae*, *P. Aeruginosa* and *S. Aureus*, respectively. This demonstrate its effectiveness in balancing precision and recall. On the other side, the SVC model also showed strong performance, particularly in multi-label scenarios (Table 7). While the XGB model showed consistent performance, its WF1 scores were generally lower compared to MLP and SVC. The RF model consistently showed the lowest WF1, indicating its relative ineffectiveness in this context (Table 7).

MLP and SVC multi-label models achieved the best WF1 across all bacterial species (Table 7). In specific, MLP shows better results for *E. Coli*, *K. Pneumoniae* and *S. Aureus*, and in the case of SVC the best results were obtained for *E. Coli*, *K. Pneumoniae* and *P. Aeruginosa*. Besides, in cases where MLP or SVC show better results, the difference from those obtained by the competing model was only 0.01 on average. For example, in the case of *P. Aeruginosa* the WF1 was 0.86 for MLP and 0.87 for SVC, and for *S. Aureus* the WF1 was 0.88 for MLP and 0.89 for SVC.

The ACC and HL values suggest that the optimal algorithm depends considerably on the bacterial species. In detail, SVC yielded the best results for *E. Coli* and *P. Aeruginosa*, XGB for *K. Pneumoniae*, and MLP for *S. Aureus*. These findings indicate that no single model consistently outperforms the others across ACC and HL metrics and bacteria.

**Table 8:** Performance of MLP and SVC models with and without oversampling (None, Random, SMOTE-Tomek) for multi-label and single-label classification scenarios across all bacteria.

Bacteria	Model	WF1					
		Single-label			Multi-label		
		None	Random	SMOTE-Tomek	None	Random	SMOTE-Tomek
*E. Coli*	MLP	**0.87**	0.73	0.85	**0.87**	0.63	0.86
	SVC	0.86	0.82	0.83	**0.87**	0.86	0.86
*K. Pneumoniae*	MLP	**0.92**	0.91	0.91	**0.92**	0.91	**0.92**
	SVC	**0.92**	**0.92**	**0.92**	**0.92**	**0.92**	**0.92**
*P. Aeruginosa*	MLP	0.86	0.84	0.83	0.86	0.86	0.86
	SVC	0.86	0.84	0.85	**0.87**	0.86	**0.87**
*S. Aureus*	MLP	**0.90**	0.88	0.89	0.88	0.89	0.89
	SVC	0.88	0.88	0.88	0.89	0.88	0.88

Results are reported in terms of the WF1.

Significant values are in bold.

### Impact of oversampling methods

Experiments were conducted to assess the impact of oversampling methods on model’s performance. The models selected for this experiment, MLP and SVC, were those that demonstrated the best performance in the previous step. Random and SMOTE-Tomek oversampling techniques were applied to balance class distributions by equalizing the number of instances in all classes with the majority class. The hyperparameters used were those optimized in prior experiments.

The results are presented in Table 8, which follows a similar structure to Table 7, with the addition of an “Oversampling” column to specify the sampling method applied (None, Random, SMOTE-Tomek). To ensure clarity, only the WF1 score was used as the performance metric for this comparison. The selection of WF1 metric to evaluate the impact of oversampling methods was because WF1 effectively handles class imbalance.

The results showed that oversampling did not lead to significant improvements; in most cases, models without oversampling yielded better performance. Particularly, the multi-label strategy without oversampling yielded better results, while in other instances, the single-label strategy, also without oversampling, resulted in superior performance.

### External validation

In order to validate the MLP and SVC models and external validation was performed in the DRIAMS B and C databases^42^. Table 9 details the results of the trained models using data from DRIAMS A with subsequent validation performed on DRIAMS B and DRIAMS C. The WF1 was used as the performance metric, and average results were obtained following the method described in the Performance Metrics section. In most cases, the multi-label approach outperformed the single-label strategy, with the most notable differences in favor of multi-label models observed in the MLP algorithm. Furthermore, among the multi-label models, MLP consistently demonstrated higher average performance compared to SVC across all scenarios analyzed. To illustrate, for *E. Coli* the best results in DRIAMS B corresponds to MLP model with a WF1 of 0.73 (single-label) and in DRIAMS C to SVC with a WF1 of 0.77 in the multi-label approach. In the case of *S. aureus* the best models in DRIAMS B and C were obtained in the multi-label approach, with values of WF1 of 0.88 and 0.85 for SVC and MLP models, respectively. Notably, the multi-label approach outperforms the single-label except for the *E. Coli* with DRIAMS B.

**Table 9:** Results obtained from the external testing datasets, DRIAMS B and C, for the bacteria *E. Coli* and *S. Aureus*, using models trained with DRIAMS A data.

Bacteria	Database	Model	WF1	
			Single-label	Multi-label
*E. Coli*	DRIAMS B	MLP	**0.73**	0.71
	DRIAMS B	SVC	0.69	0.70
	DRIAMS C	MLP	0.72	0.72
	DRIAMS C	SVC	0.76	**0.77**
*S. Aureus*	DRIAMS B	MLP	0.62	0.73
	DRIAMS B	SVC	0.77	**0.79**
	DRIAMS C	MLP	0.81	**0.88**
	DRIAMS C	SVC	0.84	0.85

Significant values are in bold.

### Analysis of features contributions

Table 10 presents the identified potential mass (*m/z*) biomarkers for each bacterium – *E. coli*, *K. pneumoniae*, *P. aeruginosa*, and *S. aureus* – using the multi-label MLP model. The Table 10 lists the top five *m/z* ranges that were most influential in predicting antibiotic resistance for each bacterium. For *E. coli*, significant *m/z* ranges include [8440, 8459] Da and [6860, 6879] Da, while for *K. pneumoniae*, key biomarkers were found in the ranges [2120, 2139] Da and [2140, 2159] Da. *P. aeruginosa* showed important features in the ranges [4880, 4899] Da and [5780, 5799] Da. Lastly, for *S. aureus*, the model highlighted *m/z* ranges such as [2400, 2419] Da and [5020, 5039] Da.

**Table 10:** Potential mass (*m/z*) biomarkers for each bacterium using the multi-label MLP model.

Bacteria	Mass ranges (Da)
*E. Coli*	[2120, 2139], [2140, 2159], [5880, 5899], [6860, 6879], [8440, 8459]
*K. Pneumoniae*	[2120, 2139], [2140, 2159], [5060, 5079], [5400, 5419], [6800, 6819]
*P. Aeruginosa*	[4880, 4899], [5780, 5799], [6020, 6039], [6340, 6359], [6620, 6639]
*S. Aureus*	[2400, 2419], [5020, 5039], [7160, 7179], [7180, 7199], [7640, 7659]

## Discussion

This study systematically investigates specific bacteria that affect humans, aiming to predict their resistance to frequently used antibiotics. The research was based on an extensive dataset from DRIAMS^42^, involving MALDI-TOF data of bacteria with their corresponding antibiotic susceptibility.

The proposed method covers the entire machine learning lifecycle process, providing a systematic and reproducible template for projects involving multi-label contexts in MALDI-TOF data classification. This lifecycle includes data acquisition from MALDI-TOF, binning to discretize the data, and thorough data cleaning and exploration phases. Visualization techniques help discern patterns through manual inspection. Additionally, a robust validation mechanism, which separates training, validation, and test sets using pseudo-random seeds, ensures reproducibility and fair model comparisons.

To address the challenges of multi-label classification, the Label Power Set strategy was proposed. Predictive models based on Support Vector Classifier, Multi-Layer Perceptron, Random Forest, and Extreme Gradient Boosting algorithms were constructed, with a parameter optimization phase ensuring well-calibrated predictive models. Three evaluation metrics: Hamming Loss, weighted F1 Score, and Accuracy were introduced to measure models’ performance.

In this study, the independent variables represent the wavelength data from the MALDI-TOF analysis of bacteria, while each target variable represents resistance or susceptibility to a specific antibiotic. For each bacterium, AST to multiple antibiotics is provided, making this a multi-label prediction problem. The study considers four bacteria and multiple target variables, which vary in nature and quantity for each bacterium.

The primary challenge was to determine whether using multi-label classification models, which predict multiple labels simultaneously, is more effective than training multiple single-label models. The experimental results indicate that multi-label models can achieve better results under certain conditions. However, this trend is not consistent across all cases. There are circumstances where single-label models outperform multi-label models (Table 7). Although we did not find statistically significant evidence that the multi-label approach was consistently superior (p-value > 0.05), we observed certain benefits of this strategy. For instance, the multi-label approach requires training only one model, simplifying the system. In contrast, single-label systems necessitate training a separate model for each antibiotic, leading to increased complexity and re-training efforts.

On the other hand, it is important to mention that is easier to obtaining data for training single-label models compared to multi-label models. In specific, in our solution training, a multi-label model requires each data instance to have information for all considered labels. Conversely, single-label models can use instances (samples) with partial information, simplifying data collection. For our experiments, we discarded instances lacking susceptibility information for all considered labels. It is likely that single-label models built with the complete available data for each label would have performed better.

Previous studies have typically focused on a single type of bacterium, yielding limited results. Moreover^41^, reported the superiority of the multi-label strategy but only studied *S. aureus*. Our study extends this perspective by including more bacteria and antibiotics to evaluate the overall effect of multi-label classification, and by increasing the number of algorithms tested, we demonstrate that the use of MLP or SVC are better alternatives to the XGB used in^41^. Future research should involve more bacteria and antibiotics to gain a clearer understanding of the impact of these classification strategies.

In our study, we chose the Label Power Set method over alternatives like Classifier Chain and Binary Relevance due to its ability to capture the dependencies between multiple antibiotic resistances. While Binary Relevance assumes independence among labels, and Classifier Chain increases model complexity by training sequentially, LPS allows us to treat all label combinations as distinct classes, effectively modeling the correlations between antibiotics. This approach was particularly beneficial given the interrelated nature of the resistance profiles in our dataset. However, the trade-off is the increased computational cost, especially with larger label sets, highlighting the need for balancing accuracy and scalability.

In problems with class imbalance, relying on common metrics such as accuracy can provide a misleading representation of model performance. For this reason, the primary metric used in this study is F1 weighted, which offers a more nuanced evaluation in imbalanced datasets. The WF1 score is a variant of the F1-score that assigns weights to each class based on the number of instances for each class. This ensures that classes with fewer instances, often critical in medical or real-world applications, contribute proportionately to the overall score.

In a multi-class classification scenario, the WF1 score is calculated by first computing the F1-score for each class and then taking the weighted average, ensuring that the metric reflects performance across all classes in a balanced manner. While we report accuracy for completeness, metrics such as WF1, precision, and recall provide a more robust evaluation in the context of multi-label classification problems. These metrics are particularly suited for assessing the model’s ability to handle the complexity and interdependence of multiple labels, ensuring that performance is not skewed by class imbalance. The selection of these metrics is not arbitrary but stems from the nature of the problem and the structure of the dataset. In our case, WF1 is chosen as the primary metric because it better captures the intricacies of multi-label classification, where class distributions can vary significantly and balancing predictive performance across all labels is crucial.

To ensure a rigorous process, we separated the data into training, validation, and testing sets to avoid bias. Hyperparameters were systematically optimized to guarantee performance. Although, various metrics were used to quantify performance, the focus was on WF1 due to data imbalance. In all cases, the best WF1 obtained exceeded 0.85 (Table 7), demonstrating the effectiveness of using machine learning for this problem.

MLP, SVC, XGB and RF algorithms have shown good performance in these type of applications^41,42,48^. In specific, our study showed that SVC and MLP consistently yielded the best results, with similar performance metrics illustrated in Table 7. In terms of complexity, SVC is simpler and requires fewer training parameters, while MLP offers greater flexibility in providing probabilistic outputs. It would be interesting to explore additional aspects of these models, such as transfer learning, to determine if models trained on these bacteria could be useful for predicting susceptibility in new bacteria. The generalization capability of neural networks could significantly influence model selection. To illustrate, MLP and SVC were better for WF1 in both approaches single and multi-label (Table 7).

In order to evaluate the effect of oversampling methods (Random and SMOTE-Tomek) on the model’s performance, these methods were applied to MLP and SVC models, which had demonstrated superior results in previous evaluations (Table 7). The analysis showed that oversampling did not result in significant improvements (Table 8); in fact, models generally performed equally or better when no oversampling was applied. For example, in the case of *S. Aureus* where the single-label MLP model achieved 0.90 WF1 without oversampling, compared to 0.89 with SMOTE-Tomek (Table 8). This pattern persisted across other bacterial datasets, suggesting that oversampling may not enhance the WF1 score in antibiotic resistance prediction tasks (Table 8).

To assess the generalization of the trained models, we evaluated the best-performing models on external datasets, specifically using the DRIAMS B and DRIAMS C databases^42^. The MLP and SVC models were tested on the *E. coli* and *S. aureus* bacteria (Table 9). In most scenarios, the multi-label approach outperformed the single-label approach, with the only exception being the performance of the MLP single-label model for *E. coli* tested on DRIAMS B (Table 9).

The results for DRIAMS B show a decline in FW1 values compared to those reported for the validation on DRIAMS A (Table 9), which was expected given that the models were tested on a different dataset, increasing the complexity of the learning task. However, the test results on DRIAMS C are notably higher and comparable to those obtained on DRIAMS A. One possible explanation for this discrepancy is the influence of geographical origin and the equipment used for data collection, which may have introduced subtle differences in the underlying patterns, affecting model performance.

Regarding the feature contribution analysis, the potential biomarkers identified through SHAP values are shown in Table 10. In the case of *E. coli*, the ions in the range of 8440 to 8459 Da were identified as relevant, coinciding with the findings of Nakamura and colleagues^17^, who directly associate it with the ST131 lineage, one of the most predominant among multi-resistant *E. coli* isolates. For the other ions identified in *E. coli*, there are no references in literature, except for the range of 6860 Da, which has been also reported by Weis^42^. In respect to *S. aureus*, *m/z* ions in the range of 2400 to 2420 Da are highlighted, these ions are associated with the presence of the mecA gene, responsible for methicillin resistance^60^. Additionally, *m/z* ions reported in the range of 5020 to 5040 Da, have been directly associated with the MRSA variant in the study of Wei-Hsiang Ma^61^. Furthermore, for the bacteria *K. pneumoniae* and *P. aeruginosa* (Table 10), no literature references associated with the relevant ions identified by the model have been found, indicating a need for further investigation of these specific biomarkers.

Overall, the patterns emerging from Table 10 suggest that specific *m/z* ranges are recurrently important across different bacteria, which may indicate common resistance mechanisms or shared protein signatures associated with antibiotic resistance. This insight provides a valuable basis for further investigation into these biomarkers, potentially aiding in the development of rapid diagnostic tools for clinical use.

From a clinical perspective, it is important to highlight that traditional antibiotic susceptibility testing, the standard method in hospital laboratories, can take up to 72 hours to yield results. In this context, the development of analogous techniques to predict bacterial resistance or susceptibility to various antibiotics could offer a significant advantage by providing results in a much shorter time in the order of seconds. Implementing multi-label classification models in conjunction with data from mass spectrometry equipment, such as MALDI-TOF, enables rapid identification of the optimal initial treatment for a patient, even before the definitive results from bacterial cultures are available. This is particularly crucial in cases where the severity of infection demands immediate intervention. Furthermore, this approach not only accelerates the decision-making process but also has the potential to deliver personalized treatment recommendations based on mass spectrometry of the patient’s bacterial infection. 

## Conclusions

In this study, we explored the prediction of antimicrobial resistance using multi-label classification across four clinically significant bacteria: *E. coli*, *S. aureus*, *K. pneumoniae*, and *P. aeruginosa*. We evaluated the performance of multiple algorithms – MLP, SVC, RF, and XGB – under both single-label and multi-label frameworks, identifying scenarios where each approach excelled. Additionally, we assessed the impact of oversampling techniques and performed external validation using DRIAMS B and C datasets to test the generalizability of the models. Our methodology, designed for MALDI-TOF data analysis, provides a reproducible framework that includes model optimization, performance evaluation using WF1, and interpretability with SHAP analysis. These efforts contribute to understanding when and how multi-label models can offer advantages in predicting antibiotic resistance, providing insights for future research and clinical applications.

In summary, SVC and MLP models show superior WF1 scores. While multi-label models can outperform single-label models, they do not always provide better performance. Nevertheless, multi-label may be advantageous from the point of view of clinical application, since in the AST more than one antibiotic is evaluated at a time, the multi-label approach allows the prediction of resistance to more than one antibiotic at the same time. Besides, several other benefits of multi-label classification are highlighted. Multi-label models simplify the system by requiring the training of only one model, reducing complexity and re-training efforts compared to single-label models, which need a separate model for each antibiotic. Furthermore, the choice of classification strategy should consider the specific context and requirements of the application.

The findings of this study expand the understanding of multi-label classification in antibiotic resistance prediction, addressing the limitations of previous studies that focused on single bacteria. Besides, analysis of SHAP values in a multi-label model provides new insight into potential biomarkers associated with multidrug resistance. Future research may involve a broader range of bacteria and antibiotics to further elucidate the impact of multi-label classification strategies.

From a clinical perspective, the significance of this study is substantial. Traditional antibiotic susceptibility testing applied in hospitals can take up to 72 hours. In contrast, the techniques developed in this study can predict antibiotic resistance in seconds. By leveraging multi-label classification models in conjunction with mass spectrometry data, clinicians can rapidly determine the most appropriate initial treatment while awaiting definitive results from bacterial cultures. This swift response is crucial in urgent medical cases requiring immediate intervention, offering not only faster but also potentially personalized treatment based on the bacterium’s MALDI-TOF MS data.

In summary, the proposed multi-label classification approach holds significant clinical relevance, as it closely mirrors the processes used in antimicrobial susceptibility testing performed in laboratories. The results demonstrate that this strategy provides competitive performance compared to the traditional single-label approach. Additionally, the multi-label framework naturally captures the intricate and interdependent nature of antimicrobial resistance data, making it a suitable choice for modeling complex resistance patterns across multiple antibiotics. These findings highlight the potential of multi-label classification to enhance the predictive capabilities in AMR studies, paving the way for future developments in clinical applications and diagnostic tools.

## Supplementary Information

Supplementary Figures.

Supplementary Tables.

## Data Availability

The datasets used and/or analysed during the current study available from the corresponding author on reasonable request. The code used to process the data and build the models is available in the following GitHub repository: Probabilistic MALDI-TOF AST.
