# Supplementary material for: Multi-label classification to predict antibiotic resistance from raw clinical MALDI-TOF mass spectrometry data
Source: Sci Rep. 2024 Dec 28;14:31283. doi: 10.1038/s41598-024-82697-w (PMC11682278; doi:10.1038/s41598-024-82697-w)
Supplement: Supplementary file 1 — Supplementary Figures. [file 41598_2024_82697_MOESM1_ESM.pdf]

Multi-label classification to predict antibiotic resistance from raw clinical MALDI-TOF mass spectrometry data.
Supplementary Material

October 21, 2024

Average Mass Spectrometry

S1 - *E. coli* Average Mass Spectrometry

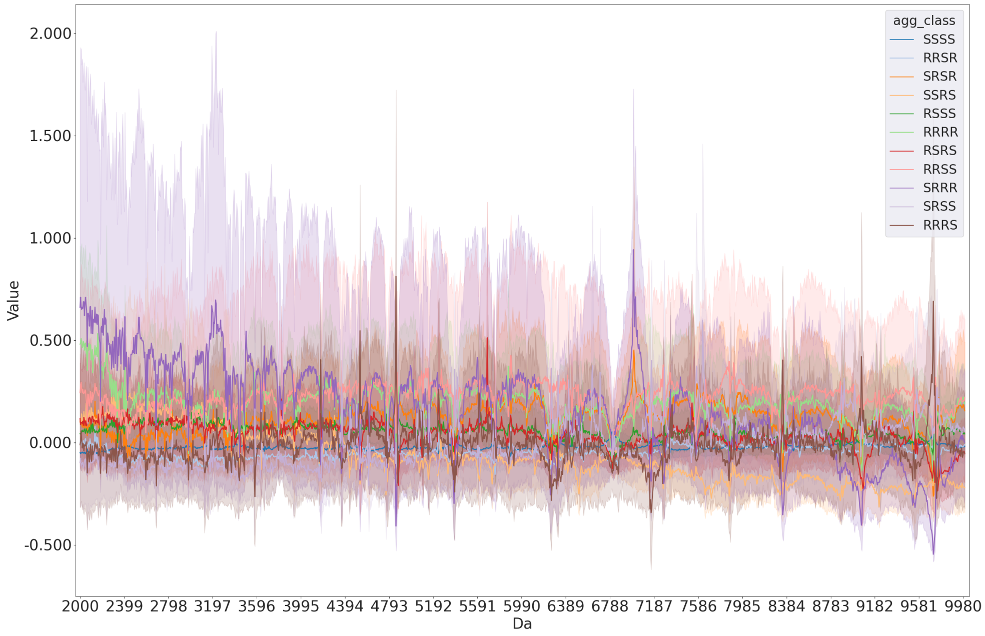

Figure 1: Average mass spectra for each antibiotic susceptibility combination of *E. coli*.

S2 - ***K. pneumoniae*** Average Mass Spectrometry

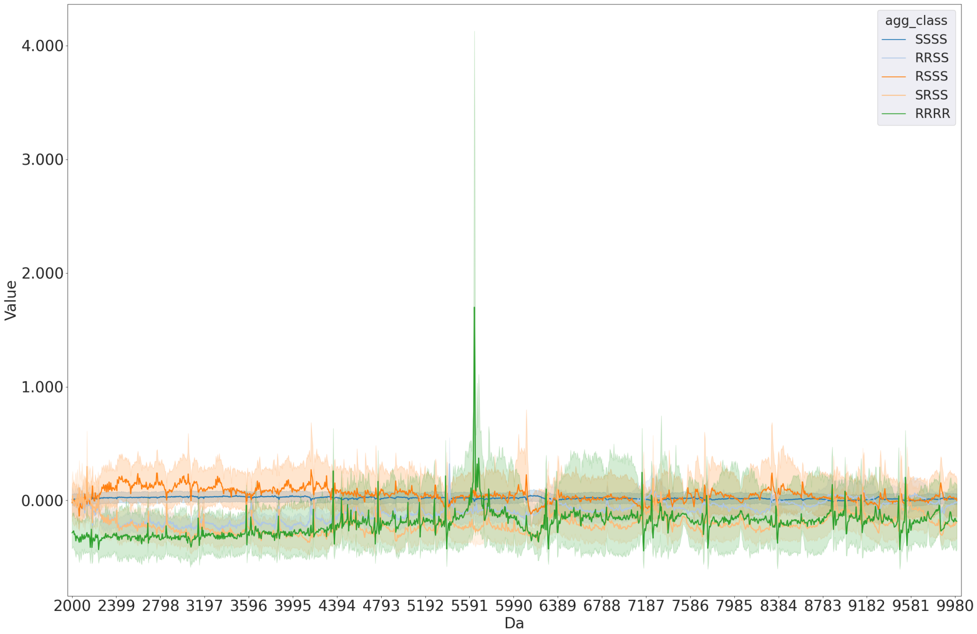

Figure 2: Average mass spectra for each antibiotic susceptibility combination of *K. pneumoniae*.

S3 - *P. aeruginosa* Average Mass Spectrometry

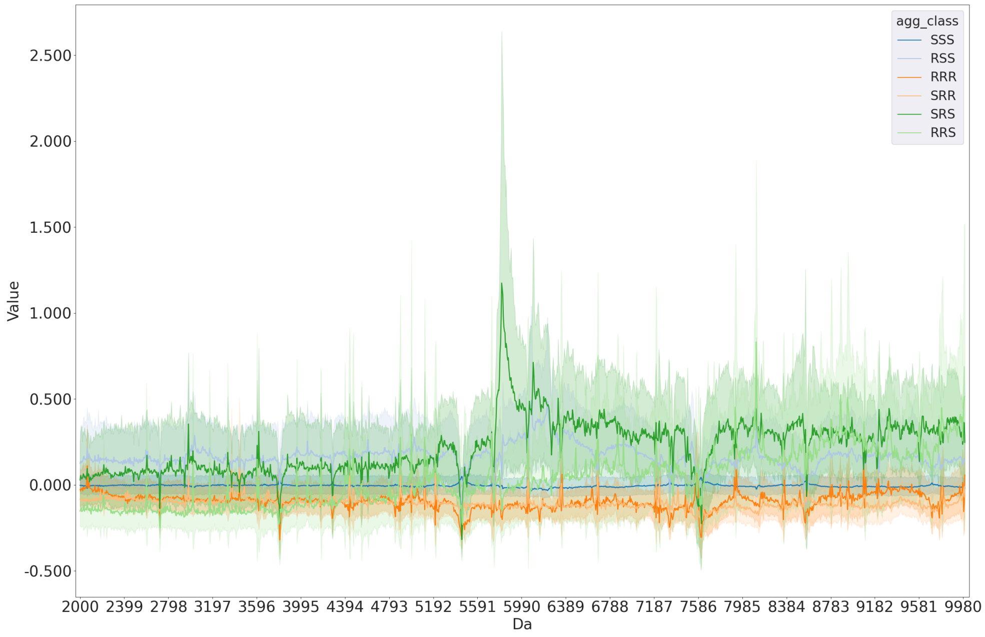

Figure 3: Average mass spectra for each antibiotic susceptibility combination of *P. aeruginosa*.

PCA

S4 - *E. coli* PCA

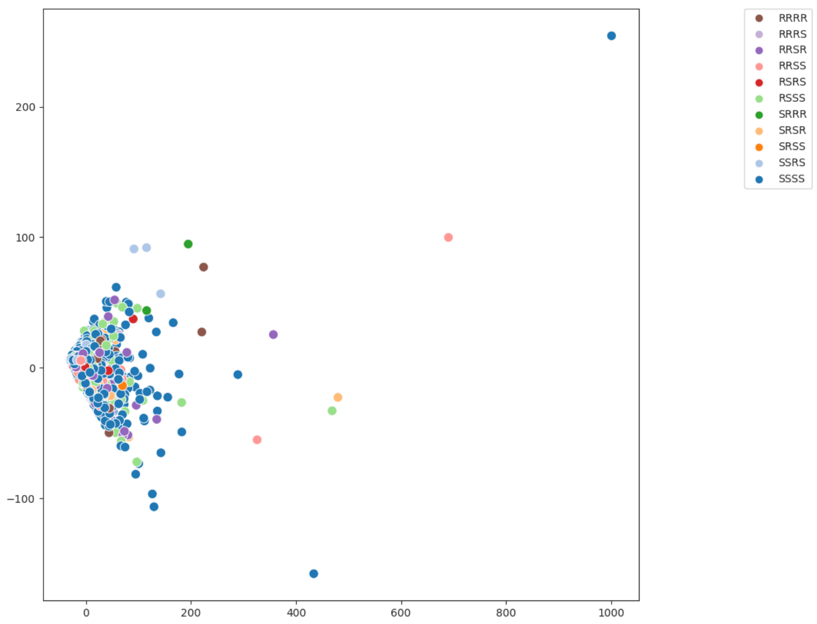

Figure 4: PCA for *E. coli* and all its AMR profiles.

S5 - *K. pneumoniae* PCA

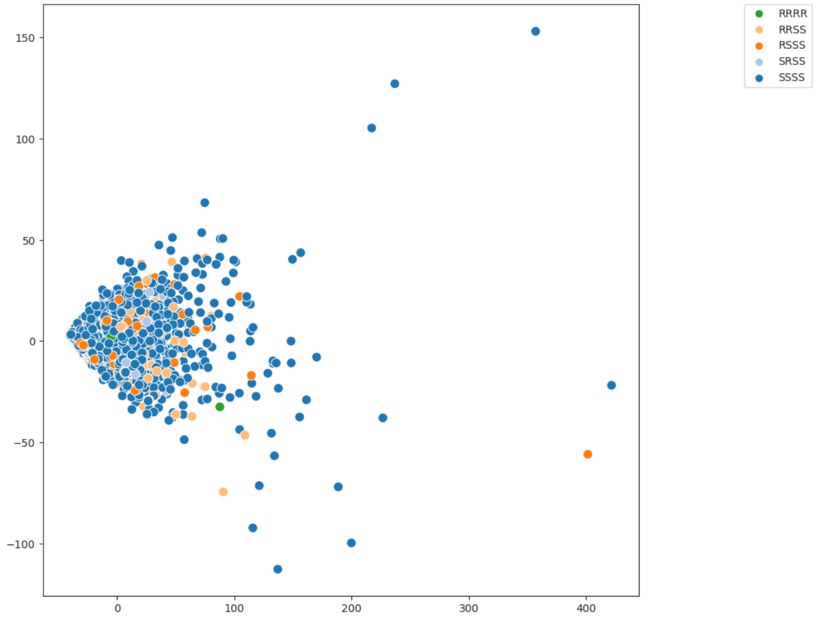

Figure 5: PCA for *K. pneumoniae* and all its AMR profiles.

S6 - *P. aeruginosa* PCA

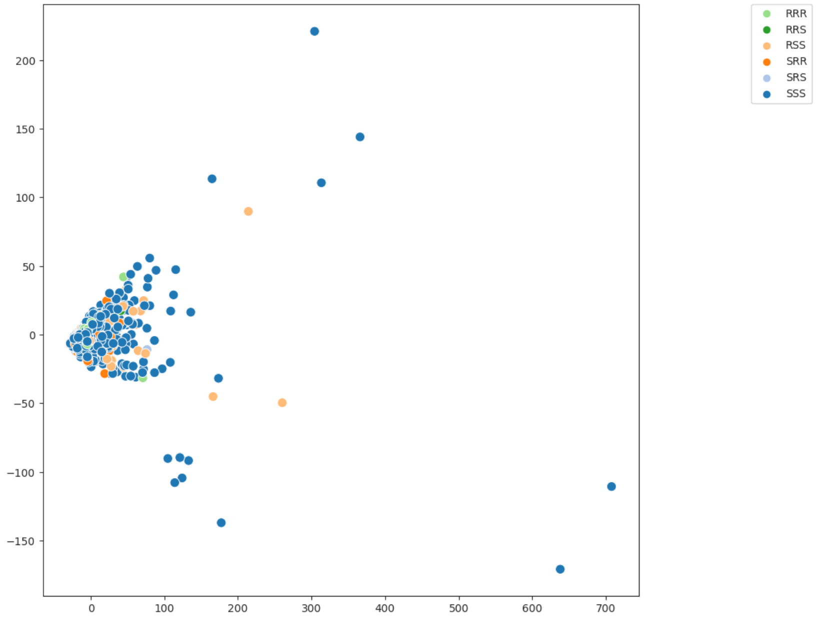

Figure 6: PCA for *P. aeruginosa* and all its AMR profiles.

T-SNE

S7 - *S. aureus* TSNE

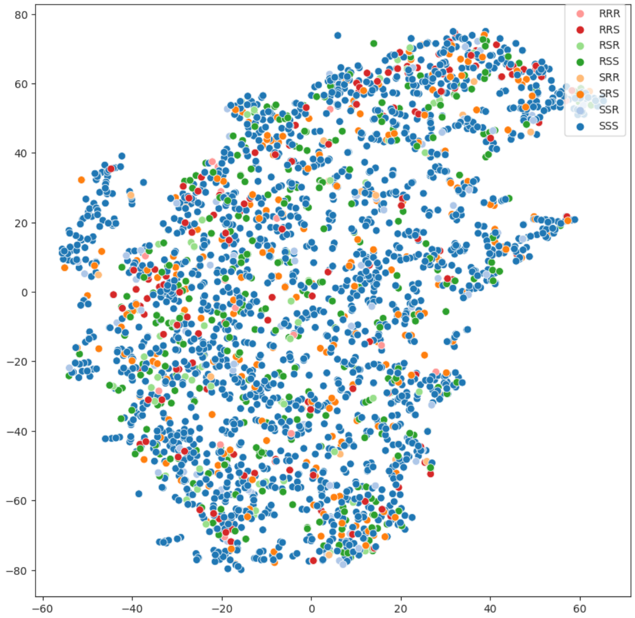

Figure 7: T-SNE for *S. aureus* and all its AMR profiles.

S8 - *E. coli* TSNE

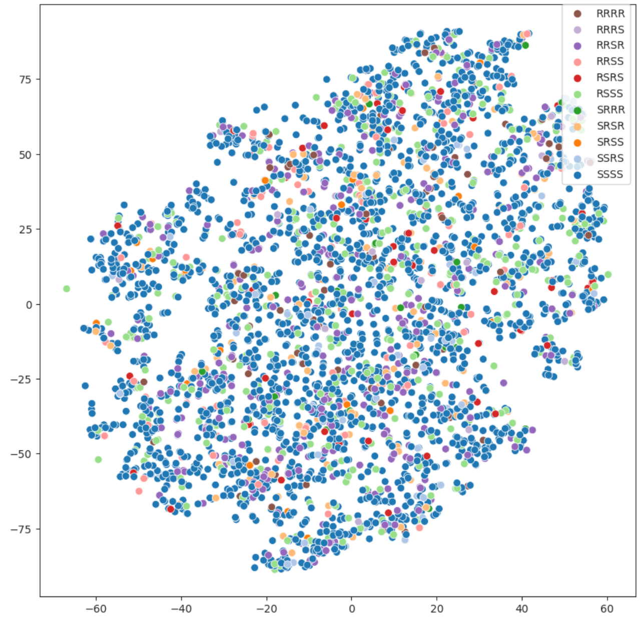

Figure 8: T-SNE for *E. coli* and all its AMR profiles.

S9 - *K. pneumoniae* TSNE

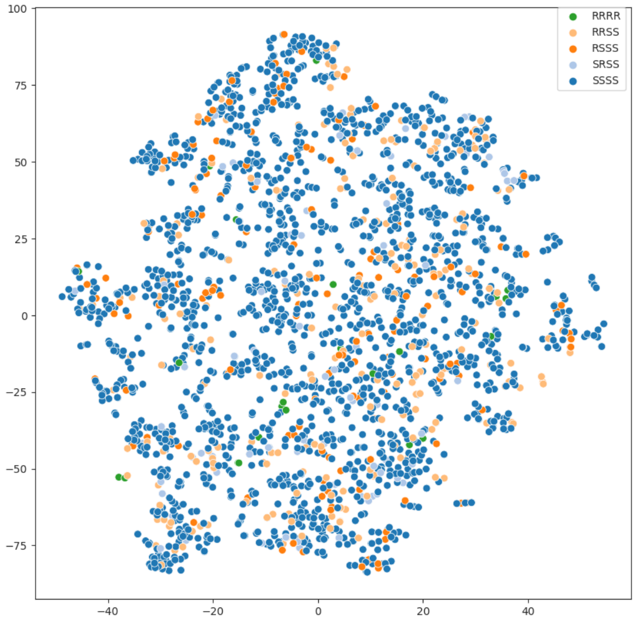

Figure 9: T-SNE for *K. pneumoniae* and all its AMR profiles.

S10 - *P. aeruginosa* TSNE

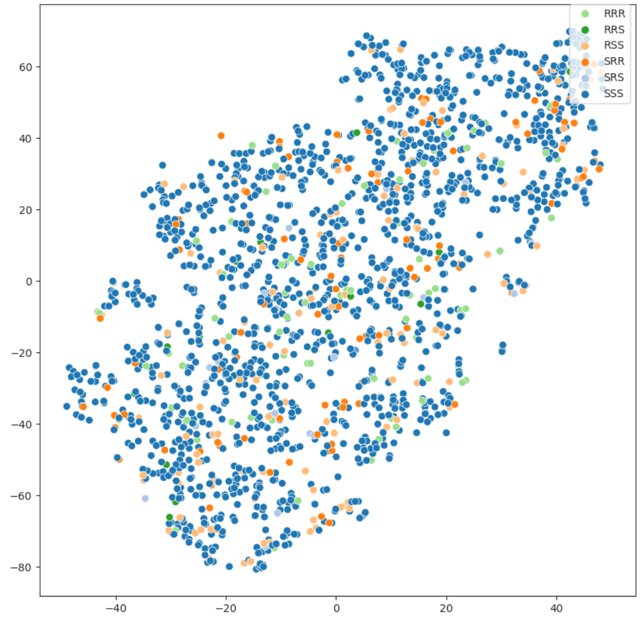

Figure 10: T-SNE for *P. aeruginosa* and all its AMR profiles.

SHAP

S11 - *S. aureus* SHAP

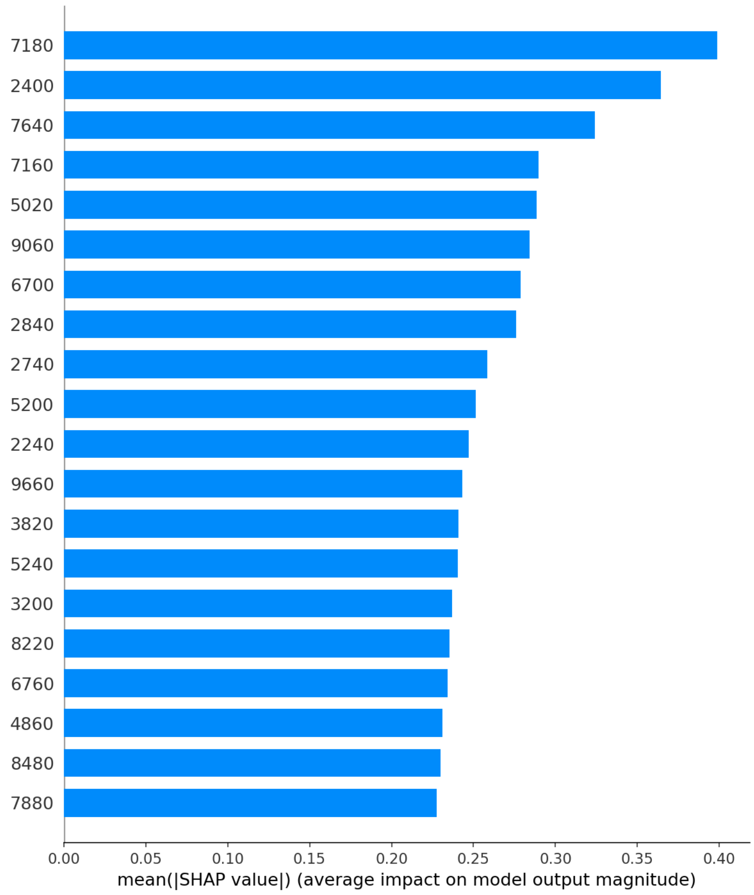

Figure 11: Most important features for the multi-label MLP model created for *S. aureus*.

S12 - *E. coli* SHAP

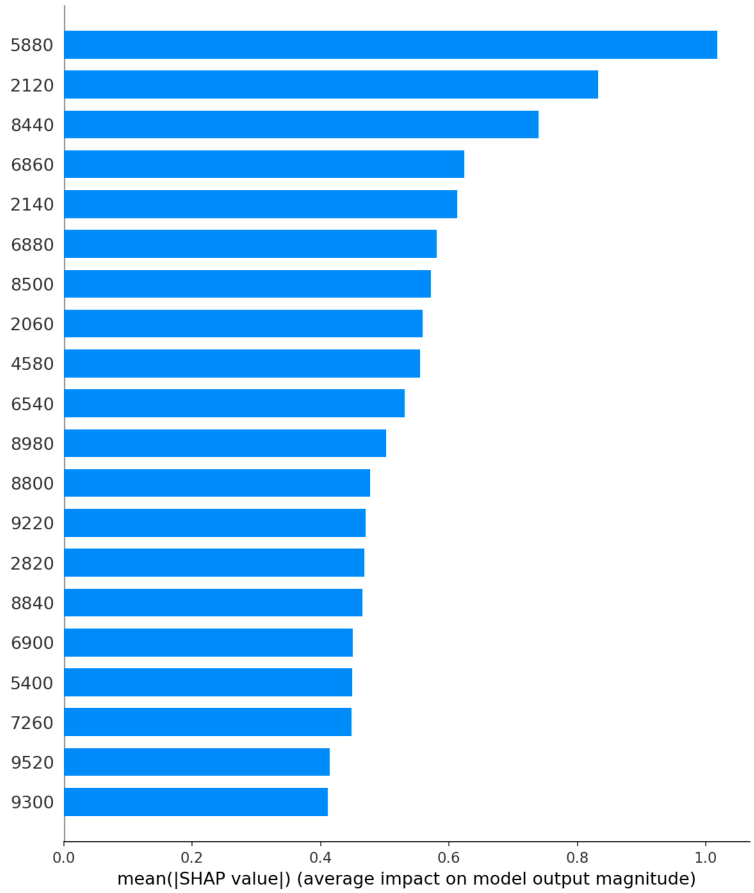

Figure 12: Most important features for the multi-label MLP model created for *E. coli*.

S13 - *K. pneumoniae* SHAP

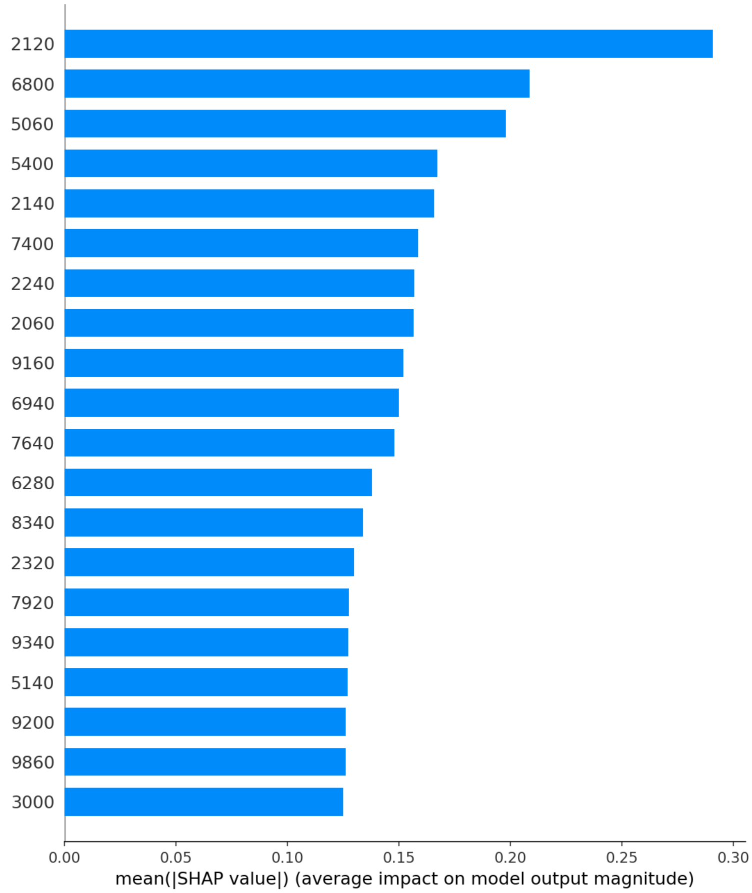

Figure 13: Most important features for the multi-label MLP model created for *K. pneumoniae*.

S14 - *P. aeruginosa* SHAP

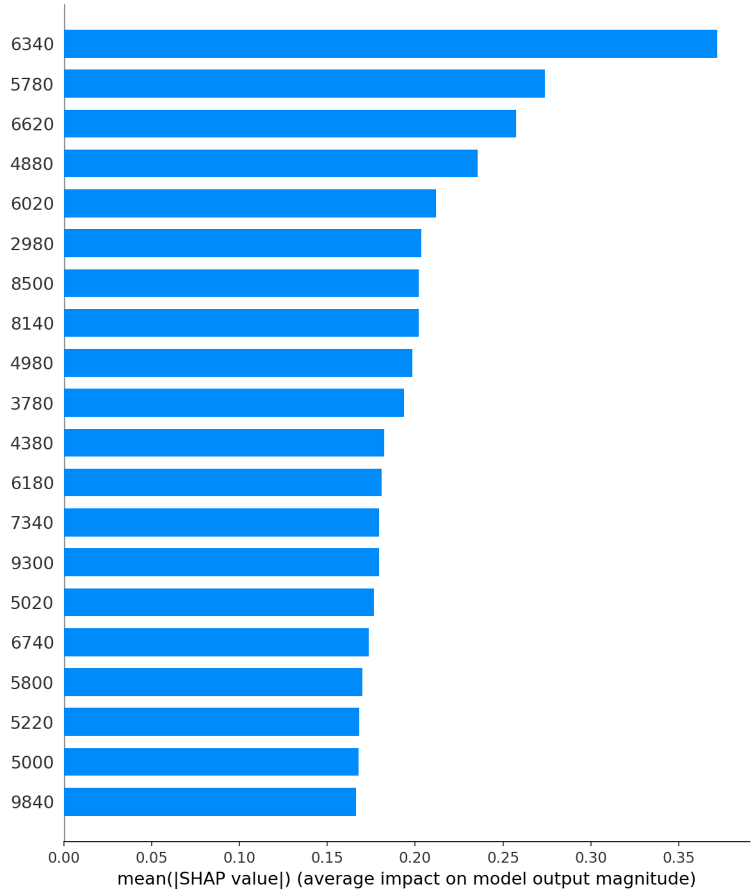

Figure 14: Most important features for the multi-label MLP model created for *P. aeruginosa*.
