# Supplementary material for: Multi-label classification to predict antibiotic resistance from raw clinical MALDI-TOF mass spectrometry data
Source: Sci Rep. 2024 Dec 28;14:31283. doi: 10.1038/s41598-024-82697-w (PMC11682278; doi:10.1038/s41598-024-82697-w)
Supplement: Supplementary file 2 — Supplementary Tables. [file 41598_2024_82697_MOESM2_ESM.pdf]

Multi-label classification to predict antibiotic resistance from raw clinical MALDI-TOF mass spectrometry data.

Supplementary Tables

October 21, 2024

Antibiotic Class Distribution

ST1 - E. coli Antibiotic Class Distribution

Ciprofloxacin	Ceftriaxone	Piperacillin-Tazobactam	Cefepime	Count
S	S	S	S	2388
R	S	S	S	421
R	R	S	R	393
S	R	S	R	115
R	R	S	S	92
R	R	R	R	81
S	S	R	S	77
R	S	R	S	53
S	R	S	S	29
S	R	R	R	25
R	R	R	S	17
S	S	S	R	0
S	R	R	S	0
S	S	R	R	0
R	S	R	R	0
R	S	S	R	0

Table 1: Class distribution for *E. coli* and its antibiotics.

ST2 - *K. pneumoniae* Antibiotic Class Distribution

Ciprofloxacin	Ceftriaxone	Imipenem	Meropenem	Count
S	S	S	S	1749
R	R	S	S	222
R	S	S	S	152
S	R	S	S	74
R	R	R	R	23
S	S	R	R	0
S	S	R	S	0
S	S	S	R	0
S	R	R	R	0
S	R	R	S	0
S	R	S	R	0
R	S	S	R	0
R	S	R	R	0
R	S	R	S	0
R	R	S	R	0
R	R	R	S	0

Table 2: Class distribution for *K. pneumoniae* and its antibiotics.

ST3 - *P. aeruginosa* Antibiotic Class Distribution

Ciprofloxacin	Imipenem	Meropenem	Count
S	S	S	1438
R	S	S	127
S	R	R	103
R	R	R	84
S	R	S	22
R	R	S	14
S	S	R	0
R	S	R	0

Table 3: Class distribution for *P. aeruginosa* and its antibiotics.
